# Cytochrome b5 reductase 2 is a novel candidate tumor suppressor gene frequently inactivated by promoter hypermethylation in human nasopharyngeal carcinoma

**DOI:** 10.1007/s13277-013-1497-1

**Published:** 2013-12-12

**Authors:** Xue Xiao, Weilin Zhao, Fangyun Tian, Xiaoying Zhou, Jinyan Zhang, Tingting Huang, Bo Hou, Chunping Du, Shumin Wang, Yingxi Mo, Nana Yu, Shiping Zhou, Jinping You, Zhe Zhang, Guangwu Huang, Xianjie Zeng

**Affiliations:** 1 Department of Otolaryngology—Head and Neck Surgery, First Affiliated Hospital of Guangxi Medical University, 6# Shuangyong Road, Nanning, 530021 China; 2 Department of Environmental and Molecular Medicine, Mie University Graduate School of Medicine, Tsu, Japan; 3 Department of Head and Neck Surgery, Affiliated Cancer Hospital of Guangxi Medical University, 71# Hedi Road, Nanning, 530021 China

**Keywords:** *CYB5R2*, Tumor suppressor gene, Inactivation, Promoter hypermethylation, Nasopharyngeal carcinoma

## Abstract

Cytochrome b5 reductase 2 (CYB5R2), a member of the flavoprotein pyridine nucleotide cytochrome reductase family, is associated with a number of physiological reactions. However, its role in cancer, especially nasopharyngeal carcinoma (NPC), has not been addressed. Here, we investigate the transcript levels and promoter methylation status of *CYB5R2* in NPC derived cell lines and tumor biopsies and experimentally address its role as a tumor suppressor gene. We find that *CYB5R2* transcript levels are decreased in NPC cell lines and tumor biopsies. Promoter hypermethylation of *CYB5R2* was detected in all six tested NPC cell lines and in 84 % of primary NPC tumor biopsies but not in normal nasopharyngeal epithelium. Clinically, *CYB5R2* methylation was associated with lymph node metastasis in NPC patients (*P* < 0.05). The endogenous expression of *CYB5R2* could be restored in vitro by the methyltransferase inhibitor 5-aza-2′-deoxycytidine in NPC cell lines. Ectopic expression of *CYB5R2* had an inhibitory effect on proliferation, clonogenicity and migration of NPC cells. Moreover, in vivo tests in nude mice indicated that ectopic expression of *CYB5R2* reduces the tumorigenicity of *CYB5R2*-negative NPC cells. Collectively, these findings suggest that *CYB5R2* may be a functional tumor suppressor gene, frequently inactivated by hypermethylation of its promoter in NPC. We report here the first instance of epigenetic downregulation in NPC tumor biopsies of a key enzyme, CYB5R2, which is responsible for the detoxification of environmental carcinogens. We propose the possibility of utilizing *CYB5R2* promoter methylation as a diagnostic biomarker of NPC in the future.

## Introduction

Nasopharyngeal cancer (NPC) is one of the most common head and neck malignant tumors in Southern China, with an annual incidence rate of 25–50 per 100,000 person-years, while the frequency in Caucasians in other countries is less than one case per 100,000 person-years [1, 2]. The etiology of NPC is multifactorial. Accumulated epidemiological and etiological evidence indicate that NPC develops as a result of a complex interaction between genetic factors, exposure to chemical carcinogens, and latent Epstein–Barr virus (EBV) infection [3–6]. Specially, environmental factors such as consumption of salt-preserved fish, cigarette smoking and exposure to environmental chemical pollutants have been reported to be of importance in the development of NPC [3, 7, 8]. EBV is strongly associated with NPC, and it has now been found to function as a tumor promoter rather than an initiator in the tumorigenesis of NPC. But the fact that the majority of the human population (>90 %) carry EBV suggest that host genetic factors might also contribute to the carcinogenesis of NPC, making it develop only in a small subset of the exposed population. However, the relative contribution of these factors in the pathogenesis of NPC remains to be elucidated in detail.

Recent studies demonstrate that genes for carcinogen-metabolizing enzymes may play critical roles in determining individual susceptibility to NPC. Genetic or epigenetic alterations that alter the expression or function of these genes, may decrease the efficiency of the corresponding carcinogen detoxification processes, which, in turn, may increase individual susceptibility and cancer risk. Xenobiotics can be detoxified by phase I or II biotransformation enzymes [9], and genetic polymorphisms in genes encoding biotransformation enzymes such as *CYP2E1*, *GSTM1* and *GSTT1* were shown to increase individual susceptibility to NPC [10–13].

Furthermore, growing evidence demonstrates that aberrant epigenetic changes, especially promoter hypermethylation of tumor suppressor genes (TSGs), are important in the multistep carcinogenesis of NPC [14, 15]. However, no studies have looked into the association of epigenetic alterations in xenobiotic-metabolizing enzymes and NPC.

The human CYB5R2 (cytochrome b5 reductase 2), a 276-amino-acid protein, contains one ferredoxin reductase-type flavin adenine dinucleotide-binding domain and belongs to the flavoprotein pyridine nucleotide cytochrome reductase family. It is one of the phase I xenobiotic biotransformation enzymes responsible for detoxification of polycyclic aromatic hydrocarbons and arylhydroxylamine carcinogens, commonly found in cigarette smoke and fried foods [16]. CYB5R2 is involved in various biological processes such as electron transport, oxidation–reduction, lipid metabolism, fatty acid desaturation and/or elongation, cholesterol biosynthesis, drug metabolism, and methemoglobin reduction in erythrocytes [17]. Moreover, it is responsible for reducing both NADH-dependent lucigenin chemiluminescence and 2-[4-iodophenyl]-3-[4-nitrophenyl]-5-[2, 4-disulfophenyl]-2*H*-tetrazolium monosodium salt in human spermatozoa [17].

In this study, we evaluated the transcriptional levels and methylation status of *CYB5R2* in NPC cell lines and primary tumor biopsies. We further addressed the TSG properties of *CYB5R2* in NPC by a series of in vitro and in vivo experiments.

## Materials and methods

### NPC cell lines, primary tumor biopsies and normal nasopharyngeal epithelium (NNE)

Six NPC cell lines (CNE1, CNE2, TW03, C666-1, HNE1, and HONE1) were maintained at 37 °C in the appropriate medium. In total, 70 NPC primary tumor biopsies were collected from newly diagnosed and untreated NPC patients at the department of Otolaryngology—Head and Neck Surgery, First Affiliated Hospital of Guangxi Medical University (Nanning, China), with informed consent from the donors. The diagnosis was established by experienced pathologists according to the World Health Organization (WHO) classification for NPC. Twenty-four NNE tissues obtained by tonsillectomy were included as controls. In all, 50 of the 70 NPC biopsies and 12 of the 24 NNEs were processed for DNA extraction, the rest 20 NPC and 12 NNEs were used for RNA extraction. All biopsy samples were stored in liquid nitrogen. Ethical permission for this study was granted by the Ethical Review Committee of First Affiliated Hospital of Guangxi Medical University, China.

### Semi-quantitative RT-PCR

Preparation of total RNA, first-strand synthesis of cDNA and RT-PCR was performed as described [18]. All primer sequences, annealing temperatures, cycling conditions and expected PCR product sizes are listed in Table 1. Glyceraldehyde-3-phosphate dehydrogenase (*GAPDH*) was amplified from the same cDNA sample as an internal control. The amplified PCR products were visualized after electrophoresis in 2 % agarose gels and semi-quantitative analysis was performed using Quantity One v4.4.0 software (Bio-Rad, USA).

**Table 1:** Primer sequences, product size and annealing temperature used in this study

Primers	Primer sequences	Product size (bp)	Annealing temperature
RT-PCR			
*CYB5R2*- forward	5′-CCTTGTAGGGACCCGTCCC-3′	291	66 °C
*CYB5R2*- reverse	5′-GACAGGGGTGTAAGCCCTG-3′		
*GAPDH*-forward	5′-AAGCTCACTGGCATGGCCTT-3′	375	60 °C
*GAPDH*-reverse	5′-CTCTCTTCCTCTTGTGCTCTTG-3′		
Methylation-specific PCR			
MSP-forward	5′-GGGGAGCGGGTTAGTCGTC-3′	140	65 °C
MSP-reverse	5′-GAACCCGCAAACTCGTAACGTC-3′		
USP-forward	5′-GGGGAGTGGGTTAGTTGTTG-3′	146	58 °C
USP-reverse	5′-CACCACAAACCCACAAACTC-3′		
Bisulfite sequencing PCR			
BGS-forward	5′-GGTAGGGTTGATTTAGAGTTAG-3′	301	58 °C
BGS-Reverse	5′-CTTCAATACTCCATAAATACACC-3′		

### Methylation-specific PCR (MSP) and bisulfite genomic sequencing (BGS)

The procedure for sodium bisulfite modification of DNA was performed as described [18, 19]. Bisulfite-modified DNA was amplified using MSP, with primer sets specifically detecting methylated or unmethylated alleles. PCR products were separated on agarose gels. MSP analyses were performed in duplicate. To analyze the methylation status of the targeted region in the *CYB5R2* promoter, bisulfite-treated DNA was amplified with a BGS PCR primer set. Amplified PCR products were subcloned and transformed into JM109 competent cells. Several isolated plasmid clones from each cell line or biopsy were sequenced using the BigDye terminator-cycle sequencing kit 3.0 (Applied Biosystems, USA) on an ABI 3100 sequencer.

### 5-Aza-2′-deoxycytidine (5-aza-dC) treatment

Four NPC cell lines (CNE1, CNE2, HONE1 and C666-1) were seeded into six-well plates at 2 × 10^5^ cells/well. The next day, cells were treated with the DNA methyltransferase inhibitor 5-aza-dC (Sigma) at 10 μM for 96 h, and medium with freshly added 5-aza-dC was replaced every 24 h. Total RNA was extracted for semi-quantitative RT-PCR as described.

### Vector construction and transfection

The full-length coding sequence of *CYB5R2* from Origene (USA) was subcloned into the pCMV-Tag3A vector (Stratagene, USA). The NPC cell line HONE1, which showed downregulated *CYB5R2*, was transfected with the pCMV-Tag3A-*CYB5R2* plasmid or control pCMV-Tag3A using Lipofectamine 2000 (Invitrogen, USA). Stable clones were obtained by G418 selection (400 μg/ml) for 2 weeks and maintained in 200 μg/ml G418.

### Colony formation assay

Aliquots of 1 × 10^5^ HONE1 cells were transfected as described above. After 48 h, the cells were transferred to 60-mm cell culture dishes and selected in 400 μg/ml G418 for 2 weeks. Giemsa-stained colonies were photographed and counted using the Quantity One v4.4.0 software (Bio-Rad, USA). The experiment was performed in triplicate.

### Cell proliferation assay

To determine the cell proliferation rate, stably transfected empty vector-HONE1 and *CYB5R2*-HONE1 cells were seeded into 96-well plates at 2 × 10^3^ cells per well. Cell density at different time points was measured using the vital stain 3-(4, 5-dimethylthiazol-2-yl)-2,5-diphenyltetrazolium bromide (MTT, Solarbio) and the absorbance (OD490 nm) was measured in a microplate reader (iMark, Bio-Rad, USA). Cells were cultured for 5 days, and five wells from each group were chosen as replicates every 24 h.

### Wound healing assay

Empty vector-HONE1 and *CYB5R2*-HONE1 cells (5×10^5^ per well) were seeded in six-well plates and allowed to adhere for 24 h. Confluent monolayers of cells were scratched by a sterile 1 ml micropipette tip. Photographs were taken at 0 and 24 h at the same position of the wound and the regrowth of cells into the wound area was measured. The experiment was performed in triplicate.

### In vivo tumor growth assay

Eight female 6-week-old Balb/c athymic nude mice (Experimental Animal Center of Guangxi Medical University, China) were used. The experimental protocol for tumor formation in nude mice was approved by the Ethical Review Committee of First Affiliated Hospital of Guangxi Medical University, and the committee's ethical guidelines for animal experimentation were followed. A total of 2×10^6^ HONE1 cells, stably transfected with *CYB5R2*, were subcutaneously injected into the right flank of the nude mice. An equal amount of empty vector-HONE1 cells were injected into the left flank of the mice as a control. The growth of tumors was monitored every 2 days for 2 weeks after inoculation. Tumor volume (*V*) was calculated as *V* = (*π*/6)*L* × *W* × *H*, where *L*, *W*, and *H* represent tumor diameters in three mutually orthogonal planes. The tumor was removed and weighed on day 14.

### Statistical analysis

Statistical analysis was performed using SPSS v16 (SPSS Inc., Chicago, IL, USA). Data are shown as mean ± SD. The association of promoter methylation status with clinical pathological features of NPC patients was analyzed by Pearson chi-square or Fisher's exact test. Paired *t* test was used to compare in vivo experiment groups. A *P* value <0.05 was considered statistically significant.

## Results

### Inactivation of *CYB5R2* in NPC cell lines and primary tumors

**Fig. 1:**
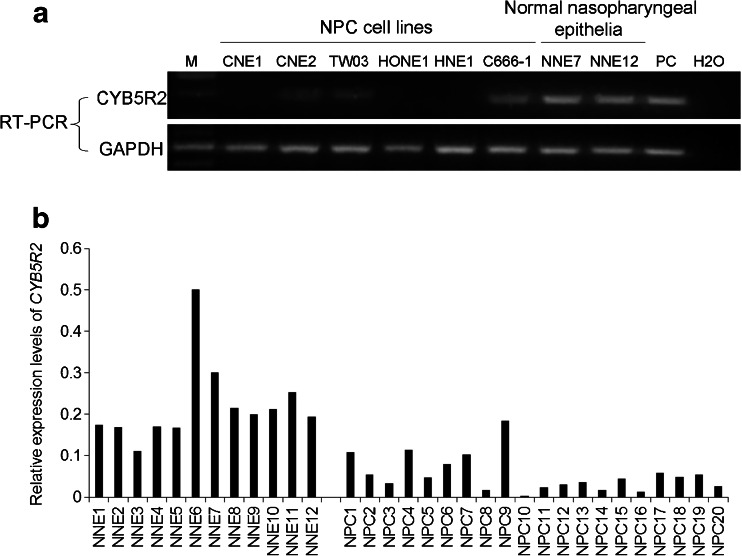
Expression of cytochrome b5 reductase 2 (*CYB5R2*) in NPC cell lines, NPC biopsies and NNE samples. **a** RT-PCR analysis of expression of *CYB5R2* in NPC cell lines and NNEs. PC: positive control. GAPDH was the internal control. Water was the blank control. **b** Semi-quantitative RT-PCR of *CYB5R2* expression in NNEs and primary NPC tumors. *CYB5R2* expression was normalized to that of GAPDH

To investigate the expression of *CYB5R2* in NPC cell lines and NPC biopsies, semi-quantitative RT-PCR was performed. As demonstrated in Fig. 1a, CY*B5R2* mRNA expression was undetectable in NPC cell lines CNE1, HONE1, and HNE1 while weak expression was detected in CNE2, TW03, and C666-1. As well, *CYB5R2* expression was downregulated in the 20 NPC primary tumor biopsies but easily detected in all NNE samples (Fig. 1b).

### The *CYB5R2* promoter is hypermethylated in NPC cell lines and primary tumors

**Fig. 2:**
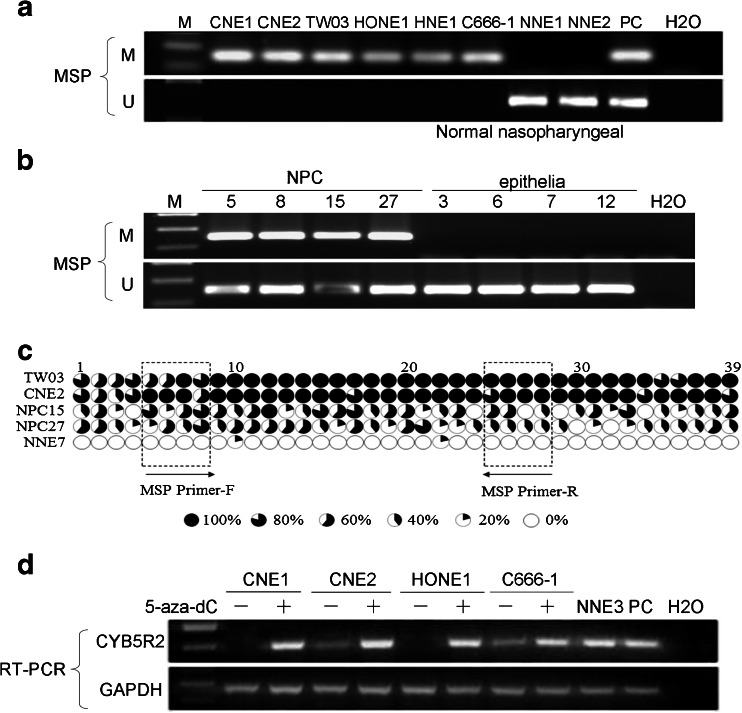
Analysis of the methylation status of the *CYB5R2* promoter region in NPC cell lines, NPC primary tumors and NNE samples. **a** MSP analysis in NPC cell lines and NNEs. *M* methylated alleles, *U* unmethylated alleles. In vitro methylated DNA was used as a methylation-positive control and DNA from normal lymphocytes was the positive control for unmethylated alleles. The blank control was water. **b** MSP analysis in NPC biopsies and NNE, representative data is shown. **c** Methylation status of the 39 CpG sites of the *CYB5R2* promoter in two NPC cell lines (TW03 and CNE2), two NPC biopsies (NPC15 and 27) and one NNE biopsy (NNE7). Five clones were randomly selected and sequenced for each sample. Different size filled sectors of the circles represent the fraction of methylated CpG. MSP primer locations are indicated by frames. **d** Restoration of *CYB5R2* expression by treatment with 5-aza-dC in NPC cell lines. One NNE sample and *CYB5R2* expression plasmid DNA were used as positive controls

Having established that *CYB5R2* was downregulated in NPC cell lines and primary biopsies, we further investigated the promoter methylation status of *CYB5R2*. MSP results showed that the *CYB5R2* promoter was hypermethylated in the six NPC cell lines (CNE1, CNE2, TW03, HONE1, HNE1 and C666-1; see Fig. 2a) and in 84 % (42/50) of NPC primary tumors but in none of the 12 NNE samples (Fig. 2b). Unmethylated amplicons were detected in some primary NPC biopsy samples but were most likely due to contaminating non-malignant cells such as stromal cells in a fraction of the tissue samples.

### CpG sites in the *CYB5R2* promoter are heavily methylated in NPC cell lines and NPC biopsies

To reveal the detailed methylation status of individual CpG sites, we analyzed the DNA sequence of the promoter region −389 to −89 bp of *CYB5R2* using BGS in two NPC cell lines (TW03 and CNE2), two NPC biopsies (NPC15 and NPC27) and one normal biopsy (NNE7) (Fig. 2c). All 39 CpG sites were heavily methylated in TW03 and CNE2, in agreement with our finding of downregulated *CYB5R2* expression in these NPC cell lines. As well, the two NPC biopsies tested showed intense methylation. The presence of some unmethylated *CYB5R2* promoter sequences among the clones originating from the two NPC cell lines TW03 and CNE2 may be due to heterogeneity in the original cell lines. The NNE sample was nearly completely free of methylation. The BGS data also demonstrate that our bisulfite conversion of genomic DNA was complete which supports the reliability of our MSP results.

### Transcription of *CYB5R2* could be restored by 5-aza-dC treatment

The expression of *CYB5R2*, which was silenced or downregulated in the NPC cell lines CNE1, CNE2, HONE1 and C666-1, could be restored after 96 h 5-aza-dC treatment (Fig. 2d). This provides support for our hypothesis that *CYB5R2* downregulation in NPC is an epigenetic event and not due to genetic rearrangement or mutation of the gene or its promoter.

### Clinico-pathological significance of *CYB5R2* promoter hypermethylation in NPC

Clinical characters of the 50 NPC patients were collected, and we found a significant association of *CYB5R2* promoter hypermethylation with the clinico-pathological variable lymph node metastasis (*P* < 0.05), however, there was no significant correlation to age, sex, cancer stage, or pathological subtypes (Table 2).

**Table 2:** Association of *CYB5R2* promoter methylation status and clinicopathological characteristics of NPC patients

	No. of patients	Promoter methylation status		*P* value
		Methylated	Unmethylated	
Age (years)				NS
< 60	37	30	7	
≥ 60	13	10	3	
Sex				NS
Male	35	28	7	
Female	15	11	4	
Cancer stage^a^				NS
I	4	3	1	
II	15	12	3	
III	17	12	5	
IV	14	12	2	
Histological subtype				NS
Keratinizing squamous cell carcinoma	7	6	1	
Non-keratinizing carcinoma	43	35	8	
Lymph node metastasis				*P* < 0.05
Presence	33	33	0	
Absence	17	10	7	

Data are number of patients

*NS* not significant

^a^According to the International Union Against Cancer (UICC)

### Reexpression of *CYB5R2* in NPC cells suppresses colony formation and cell proliferation and inhibits cell migration

**Fig. 3:**
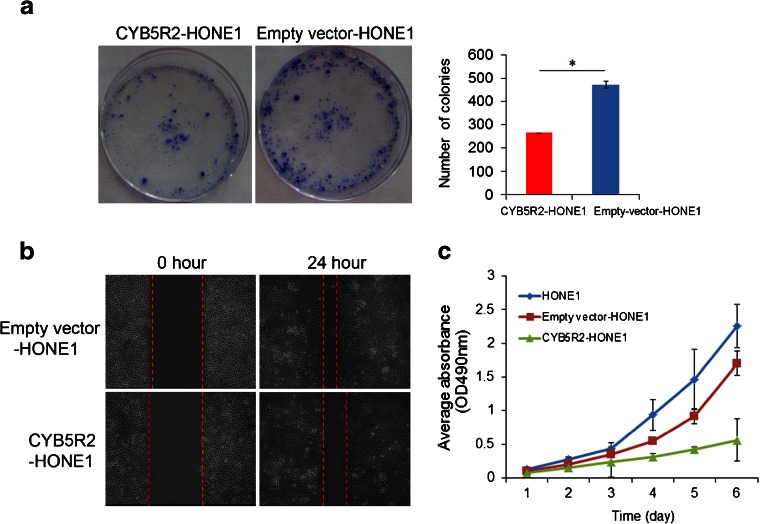
Ectopic expression of *CYB5R2* inhibits colony formation, migration and proliferation of NPC cells. **a** Colony formation ability of *CYB5R2*-transfected and empty vector-transfected HONE1cells. Colony numbers in the bar graph represent the mean ± SD of three independent experiments. **b** Migration of *CYB5R2*-transfected and empty vector-transfected HONE1cells examined by wound healing assay. **c** Growth curves of *CYB5R2*-transfected, empty vector-transfected HONE1cells and parental cells. Data are mean ± SD (*n* = 5). *P < 0.05

To assess whether *CYB5R2* might have properties of a TSG in NPC, we examined the effect of *CYB5R2* on clonogenicity and cell proliferation in HONE1 cells. The number of colonies was lower for *CYB5R2*-HONE1 than empty vector-HONE1 cells (*P* < 0.05) (Fig. 3a). As well, *CYB5R2*-HONE1 cells grew significantly slower than HONE1 parental and empty vector-HONE1 cells (Fig. 3c). To measure the effect of CYB5R2 reexpression on cell mobility, we used a wound healing assay. Cell movement into the scratched area was slower for *CYB5R2*-HONE1 than for empty vector-HONE1 cells, which suggests that ectopic expression of *CYB5R2* effects a reduction in the motility of NPC cells (Fig. 3b).

### Inactivation of CYB5R2 is critical for tumorigenesis in vivo

**Fig. 4:**
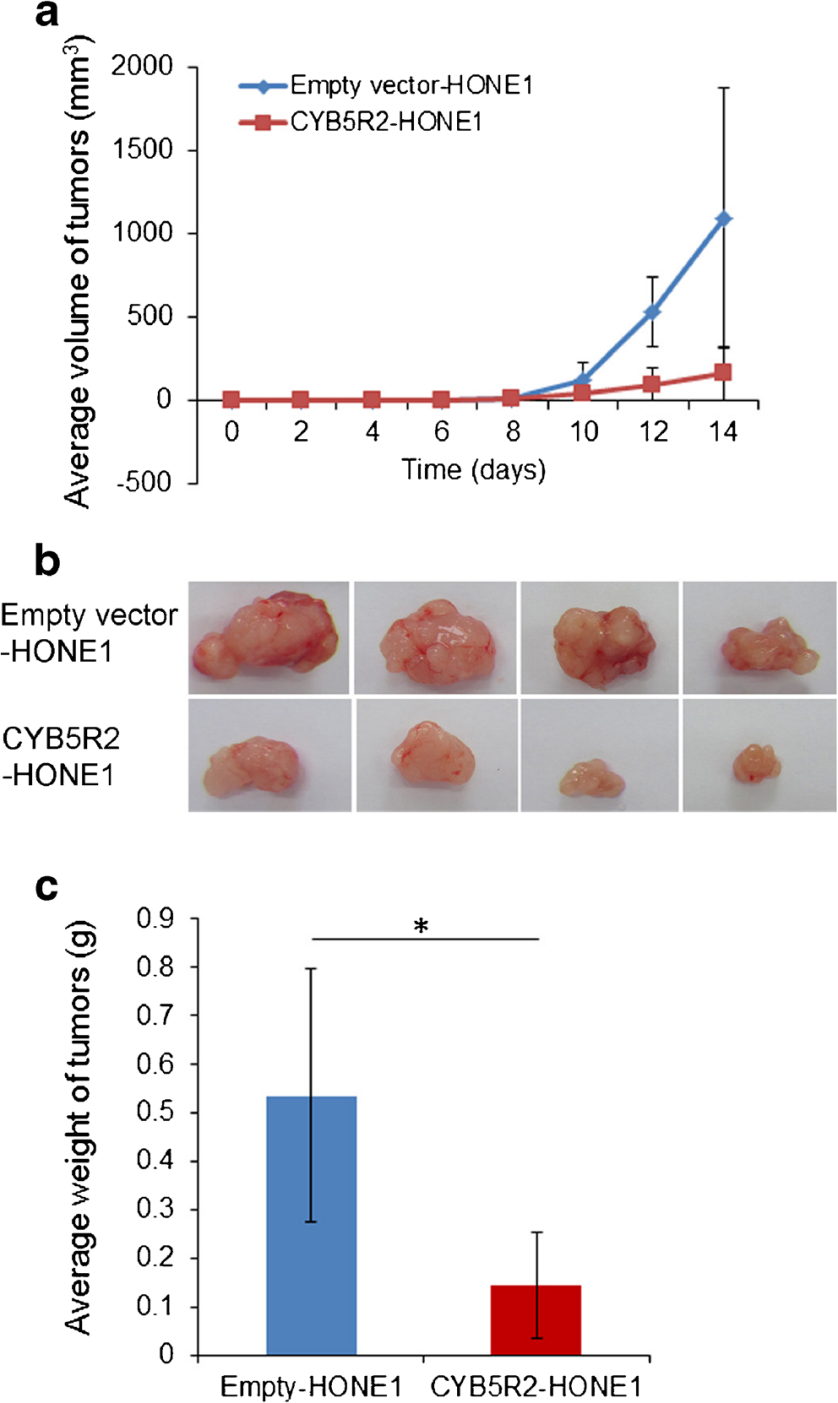
*CYB5R2* reexpression reduces tumorigenicity of NPC cells in nude mice. **a** Growth curves of tumors in nude mice. The mean volume of tumors from *CYB5R2*-HONE1 and empty vector-HONE1 cells was evaluated every 2 days after inoculation. Data are mean ± SD (*n* = 8). **b** Representative tumors were removed from the nude mice at day 14 after transplantation. **c** Mean ± SD weight of tumors (*n* = 8). *P < 0.05

We further assessed the tumor suppressing capacity of *CYB5R2* in vivo. Empty vector-HONE1 and *CYB5R2*-HONE1 cells were injected into the left and right flanks, respectively, of Balb/c athymic nude mice and tumor growth was monitored with 2-day intervals. Mice injected in the flanks with control cells formed larger tumors within 14 days, whereas mice injected in the flanks with *CYB5R2*-HONE1 showed greatly reduced tumor growth (*P* < 0.05) (Fig. 4). Thus, ectopic expression of *CYB5R2* reverses the tumor growth which is associated with its epigenetic downregulation in the HONE1 cells.

## Discussion

NPC has been proposed to be the result of a multistep process involving environmental carcinogens, genetic factors and latent EBV infection. Among these etiologic factors, EBV is a well-recognized causative factor not only for NPC, but for a number of human malignancies [20]. However, although EBV infects and persists in about 90 % of the human population, NPC develops in only a minority of EBV-infected individuals, which suggests that other risk factors may be involved in disease development. Environmental factors such as lifestyle and occupational exposure to chemical carcinogens and tumor promoters have been suggested as cofactors [3]. Examples of the most relevant environmental exposures are Cantonese-style salted fish, fermented fish sauce and other preserved foods [3, 8]. These foods contain large amounts of *N*-nitrosodimethylamine (NDMA), *N*-nitrosopyrrolidene (NPYR) and *N*-nitrosopiperidine (NPIP), which may contribute to the carcinogenic development in NPC [21]. Occupational exposure to formaldehyde and wood dust are risk factors as well [22–24]. Moreover, cigarette smoke has been consistently found as a moderate risk factor for NPC [7, 25]. Polycyclic aromatic hydrocarbons, nitrosamines and aromatic amines are known carcinogens in cigarette smoke [26, 27]. These aromatic amines can either be activated to carcinogenic aromatic hydroxylamine derivatives in the cytochrome P450 pathway or detoxified by *N*-acetylation in the cytochrome B5 reductase (CYB5R) pathway [16]. The nitrenium or nitroso derivatives produced in the cytochrome P450 activation pathway generate mutagenic DNA adducts while the end products of the CYB5R mediated detoxification pathway are cleared as *N*-acetylated, water soluble products. Alterations of genes that encode phase I and phase II xenobiotic biotransformation enzymes [9], involved in metabolic activation or detoxification of potentially carcinogenic substances, have been demonstrated as risk factors for NPC [10–13].

In the present study, we demonstrate that the mRNA expression of a xenobiotic-metabolizing enzyme, *CYB5R2*, was downregulated in NPC cell lines and primary tumors. The *CYB5R2* promoter was aberrantly methylated in all six NPC cell lines (100 %) and in 42 of 50 (84 %) primary NPC tumors, but not in any of the 12 normal controls. Bisulphite genomic sequencing demonstrated that all 39 CpG sites in the tested *CYB5R2* promoter were heavily methylated in the NPC cell lines CNE2 and TW03, where *CYB5R2* expression is downregulated. A high degree of methylation was also observed in primary NPC tumors. *CYB5R2* expression could be restored in four NPC cell lines after treatment with the demethylating agent 5-aza-dC. These results indicate that *CYB5R2* was transcriptional inactivation in NPC, and the major mechanism could be a high degree of methylation in its promoter region, which might contribute to the tumorigenesis of NPC.

The expression of *CYB5R2* has been evaluated in several human malignancies. Consistent with our findings, microarray analysis revealed that *CYB5R2* is downregulated in lobular and ductal invasive breast cancer biopsies and functions as a regulator of cell proliferation, differentiation and transformation [28]. However, these findings are inconsistent with observations in acute B lymphoblastic leukemia, in which *CYB5R2* is upregulated [29]. Thus, epigenetic inactivation of *CYB5R2* might be tissue-specific events.

Hypermethylated DNA can serve as a biomarker for cancer detection because of its high specificity in distinguishing cancers from normal tissues. We found that the *CYB5R2* promoter region was subject to methylation in all of our six NPC cell lines and 84 % of NPC biopsies. Therefore, *CYB5R2* promoter hypermethylation is a frequent event in NPC and not just a phenomenon associated with in vitro cell culture. In addition, epigenetic silencing of *CYB5R2* was a tumor-specific event and could be detected in both early- and advanced-stage NPC biopsies, which suggests that *CYB5R2* promoter hypermethylation might contribute both to the initiation and progression of NPC. *CYB5R2* methylation could serve as a potential biomarker for early diagnosis of NPC. However, due to the lack of patients' follow-up study, we suffered from the limitation that no correlation between the expression of *CYB5R2* and the survival rate of NPC patients was verified.

Lymph node metastasis is one of the most significant features of NPC [30]. It is strongly correlated to reduced survival rate and increased risk of tumor recurrence. Identification of biomarkers for lymph node metastasis in NPC would be helpful in designing optimized and individualized therapeutic regimens for NPC. Our finding that *CYB5R2* promoter methylation is associated with lymph node metastasis in NPC, suggests that *CYB5R2* promoter methylation may serve as a diagnostic indicator of lymph node metastasis, which would have great value for the clinical evaluation of NPC cases.

We show that ectopic expression of *CYB5R2* significantly inhibited cell proliferation, colony formation and migration of NPC cells in vitro. Moreover, exogenous expression of *CYB5R2* inhibits tumorigenicity of NPC cells in vivo in a mouse model system. This evidence strongly implies that *CYB5R2* is a putative TSG and plays a role in nasopharyngeal carcinogenesis. To our knowledge, this is the first report of a xenobiotic-metabolizing enzyme functioning as a tumor suppressor in NPC.

In summary, our data demonstrate for the first time that *CYB5R2* is epigenetically inactivated by promoter hypermethylation in a human cancer, NPC. Our experimental data provide support for *CYB5R2* as a novel and important candidate TSG in NPC. We revealed a frequent and tumor-specific hypermethylation of the *CYB5R2* promoter in NPC and a significant association of *CYB5R2* promoter hypermethylation with lymph node metastasis, which suggests that the epigenetic state of the *CYB5R2* promoter may serve as a diagnostic biomarker and present a possible therapeutic target for NPC.
